# Drug-resistant bacteria in the critically ill: patterns and mechanisms of resistance and potential remedies

**DOI:** 10.3389/frabi.2023.1145190

**Published:** 2023-06-30

**Authors:** Riaz M. Karukappadath, Dumitru Sirbu, Ahmed Zaky

**Affiliations:** ^1^ Department of Anesthesiology and Perioperative Medicine, University of Alabama at Birmingham, Birmingham, AL, United States; ^2^ Department of Pharmacology, Ascension St. Vincent’s, Birmingham, AL, United States

**Keywords:** MRSA, antibiotic resistance, antibiotic stewardship, ESBL, multidrug-resistant bacteria

## Abstract

Antimicrobial resistance in the intensive care unit is an ongoing global healthcare concern associated with high mortality and morbidity rates and high healthcare costs. Select groups of bacterial pathogens express different mechanisms of antimicrobial resistance. Clinicians face challenges in managing patients with multidrug-resistant bacteria in the form of a limited pool of available antibiotics, slow and potentially inaccurate conventional diagnostic microbial modalities, mimicry of non-infective conditions with infective syndromes, and the confounding of the clinical picture of organ dysfunction associated with sepsis with postoperative surgical complications such as hemorrhage and fluid shifts. Potential remedies for antimicrobial resistance include specific surveillance, adequate and systematic antibiotic stewardship, use of pharmacokinetic and pharmacodynamic techniques of therapy, and antimicrobial monitoring and adequate employment of infection control policies. Novel techniques of combating antimicrobial resistance include the use of aerosolized antibiotics for lung infections, the restoration of gut microflora using fecal transplantation, and orally administered probiotics. Newer antibiotics are urgently needed as part of the armamentarium against multidrug-resistant bacteria. In this review we discuss mechanisms and patterns of microbial resistance in a select group of drug-resistant bacteria, and preventive and remedial measures for combating antibiotic resistance in the critically ill.

## Introduction

Antimicrobial resistance in the ICU (intensive care unit) is a growing healthcare concern. The Centers for Disease Control and Prevention (CDC) estimates that more than 2.8 million antibiotic-resistant infections occur each year, with more than 35,000 deaths as a result (Despotovic et al., 2020). Continued exposure to antibiotics is one of the most important factors, if not the most important factor, in the development of antimicrobial resistance (Bell et al., 2014). In the ICU, infections due to multidrug-resistant (MDR) bacteria lead to poor clinical outcomes, prolonged ICU and hospital stays, high mortality rates, and higher healthcare costs (Campion and Scully, 2018).

A set of nosocomial pathogens, colloquially termed ESKAPE organisms, are of particular interest because of their propensity for antimicrobial resistance; these organisms are *Enterococcus faecium*, *Staphylococcus aureus*, *Klebsiella pneumoniae*, *Acinetobacter baumannii*, *Pseudomonas aeruginosa*, and *Enterobacter* species (Rice, 2008). ICU providers face challenges in diagnosing and treating MDR bacteria in the form of differentiating colonization from infection, mimicry of sepsis by non-infective syndromes such as heart failure and inflammatory conditions, the slowness and inaccuracy of current diagnostic modalities of bacterial infections in terms of Gram stains and microbial cultures, and the confounding of signs of organ system dysfunction in sepsis by postoperative complications such as hypotension and hemorrhage (Lam et al., 2018; Kisat and Zarzaur, 2022). Although previous reports have focused on specific genres of either bacteria or antibiotic resistance, this report provides a more comprehensive approach to the problem of antimicrobial resistance in a sample of Gram-positive and Gram-negative bacteria. In this report we will discuss risk factors and mechanisms of antimicrobial resistance with a focus on a group of resistant bacteria frequently encountered in the ICU. We will also provide an update on current and future preventive and therapeutic measures to manage antimicrobial resistance.

## Risk factors for acquiring MDR bacteria in the ICU

Risk factors for acquiring MDR bacteria in the ICU can be classified as patient, epidemiological, or geographic factors. Patient factors include an age of more than 60 years; diabetes mellitus; immunosuppression; a history of use of third-generation cephalosporins, vancomycin, or corticosteroids; and chronic liver or kidney disease. Epidemiological factors include duration of hospitalization, use of invasive catheters, hemodialysis, and high colonization pressure in the community. Geographical factors include travel from one geographic area with a high prevalence of MDR to another area (Burillo et al., 2019). Several scoring systems have been developed to predict, and stratify by risk, patients who are prone to become infected by MDR with variable sensitivity, specificity, and predictive values (Vasudevan et al., 2014; Teysseyre et al., 2019).

## Types and mechanisms of antimicrobial resistance

Antimicrobial resistance can be broadly categorized into two distinct groups: natural and acquired. Acquired resistance can be a consequence of repeated exposure to antibiotic therapy, leading to gene mutations within a population of bacteria. It can also come about through a phenomenon called horizontal gene transfer (HGT). Indeed, HGT is the single-most important determinant of high-level antibiotic resistance in ICUs around the world and is directly correlated to antibiotic exposure (Baditoiu et al., 2017). Although it is true that genetic mutations impart resistance to a single class of antibiotics, it would take an innumerable amount of time for enough mutations to accumulate and produce resistance to multiple classes within the same organism. HGT bypasses this mechanism via the exchange of genetic material (i.e., DNA) encoding various resistance genes, giving rise to multidrug-resistant (MDR) organisms. ESKAPE organisms are a notable set of nosocomial pathogens because of their ability to evade and adapt to antimicrobial exposure through various modes of resistance (De Oliveira et al., 2020; Prajapati et al., 2021).

**Figure 1:**
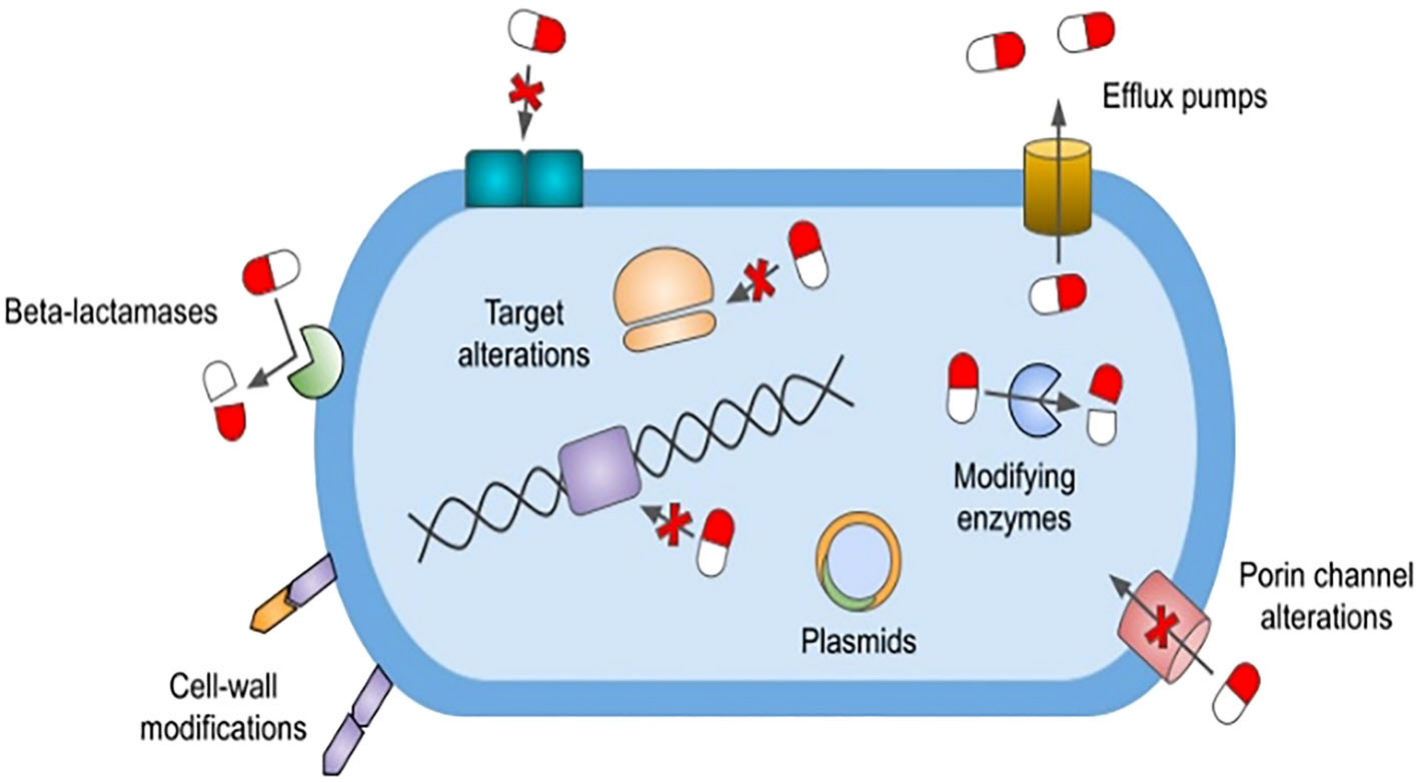
Mechanisms of antimicrobial resistance. The figure depicts various antimicrobial resistance strategies utilized by ESKAPE pathogens. β-lactamases confer various levels of resistance to β-lactam antibiotics and are classified as Amber’s class A, B, C, and D. Cell wall modifications alter the synthesis of lipopolysaccharide lipid A or peptidoglycan precursors, thereby preventing the inhibition of cell wall synthesis. Bacteria can also develop antimicrobial resistance by altering target binding sites via genetic mutations. These alterations include modification to penicillin-binding proteins, alterations to DNA gyrase and topoisomerase IV enzymes, and methyltransferase-mediated modifications to the 30S and 50S ribosomal subunits. Outer membrane porin channel alterations prohibit entry of various antimicrobials into the cell. The expression of transmembrane efflux pumps, classified into six major families, allows bacteria to actively remove nearly all classes of antibiotics from their cytoplasm. Plasmids are small mobile genetic elements composed of extrachromosomal DNA. They can spread resistance genes between bacteria of the same or different species, with the plasmid-mediated quinolone resistance gene being one such example. *ESKAPE pathogens: *Enterococcus faecium, Staphylococcus aureus, Klebsiella pneumoniae, Acinetobacter baumannii, Pseudomonas aeruginosa*, and *Enterobacter* species.

Natural resistance is defined as a genetically encoded trait that is shared by the entire population of a species regardless of antibiotic exposure. For example, vancomycin is a large glycopeptide antibiotic with activity against Gram-positive organisms that inhibits cell wall synthesis by binding the terminal d-Ala, d-Ala of the peptidoglycan precursor.^9^ Gram-negative bacteria are naturally resistant to the effects of vancomycin owing to the limited permeability of their outer cell wall to this large molecule (https://paperpile.com/c/MbHZBP/CZmw). Another well-documented example is the propensity of members of the genus *Enterococcus* to have a naturally occurring high level of cephalosporin resistance due in part to an expression of low-affinity penicillin-binding proteins (PBPs), notably PBP5 (Rice et al., 2009). In addition, natural resistance may also manifest itself as a spontaneous genetic mutation, granting resistance to an organism that is usually susceptible to a particular agent. For example, *Escherichia coli* has been shown to acquire resistance to quinolones through mutations in the quinolone resistance-determining regions of the *gyrA* and *parC* genes (Zeng et al., 2020) (**Figure 1**).

## Examples of drug-resistant bacteria

1. *Enterococcus faecium* isolates can be pathogenic and have a tendency to be highly resistant to multiple classes of antibiotics, including β-lactams (especially cephalosporins), aminoglycosides, and folate reductase inhibitors (i.e., sulfamethoxazole/trimethoprim) (Rosselli Del Turco et al., 2021). An appreciable increase in ampicillin-resistant *Enterococcus* spp. within U.S. hospitals was noted to occur in the late 1980s and was mainly driven by the widespread use of penicillin in the preceding decades.^19^ The predominant mechanism of resistance was found to be the development of low-affinity PBPs, specifically PBP5 (Leclercq et al., 1988). Around the same time period, the use of vancomycin was on the rise because of an increase in methicillin-resistant *Staphylococcus aureus* (MRSA) isolates (D'Agata et al., 2009). Vancomycin-resistant *Enterococcus* (VRE) isolates were first reported in England in 1988, followed by rapid identification in other parts of Europe and the United States. Resistance to vancomycin is conferred by the *vanA*, *vanB*, and *vanC* genes, which are found within various species of *Enterococcus*. The inducible *vanA* and *vanB* transposons have the propensity for horizontal transfer between various *Enterococcus* species, whereas the *vanC* gene is an intrinsic resistance mechanism found in *Enterococcus gallinarum* and *Enterococcus casseliflavus (* Arthur and Courvalin, 1993). Although the incidence of VRE infections between 2012 and 2017 decreased, the CDC expects the VRE burden to reach 54,500 cases and lead to 5,400 deaths annually (Reyes et al., 2016).

2. *Staphylococcus aureus* is of particular concern owing to its ability to develop methicillin resistance via acquisition of the staphylococcal cassette chromosome *mec*, which carries the resistance gene *mecA (* Hardy et al., 2004). Since the discovery of MRSA in the 1960s it has been a pathogen of interest for both community- and hospital-acquired infections. A 2005 report by Kuehnert et al. showed that there were 291,542 hospital discharges with *S. aureus*-related-infection diagnoses between 1999 and 2000. They also reported the overall rate of methicillin resistance for *S. aureus* to be 43.2% (Kuehnert et al., 2005). More recently, a French study looked at the impact of antibiotic exposure and selection of MRSA in hospital settings. They found that a more prolonged antibiotic exposure in ICUs than in general wards promoted the dissemination of MRSA in the hospital (Patry et al., 2008). In addition, other studies have corroborated the finding that excessive antibiotic exposure promotes the emergence of MRSA infections (Kardas-Sloma et al., 2011). Despite the high utilization rates of antibiotics in ICUs across the nation, a 2019 CDC report reported a 74% drop in the incidence of hospital-acquired MRSA bloodstream infections between 2005 and 2016, with an estimated 119,247 cases of *S. aureus* bloodstream infections and 19,832 associated deaths in 2017 (Wunderink et al., 2020).

3. *Klebsiella pneumoniae* is a commensal organism, normally found in the human intestine, and an opportunistic pathogen that can cause a range of infections. The 2019 CDC antimicrobial resistance threat report lists carbapenem-resistant *Enterobacterales* (CRE) as an urgent threat and extended-spectrum β-lactamase (ESBL)-producing *Enterobacterales* as a serious threat, with 13,100 estimated cases and 1,100 estimated deaths in hospitalized patients and 197,400 estimated cases and 9,100 estimated deaths in hospitalized patients, respectively, that year (CDC 2022). *K. pneumoniae* can express a multitude of resistance genes rendering a wide range of β-lactams, including penicillins, broad-spectrum cephalosporins, and carbapenems, ineffective in its treatment. Resistance to penicillins and early-generation cephalosporins can arise from mutations of genes encoding TEM-1, TEM-2, or SHV-1, whereas resistance to third-generation cephalosporins is encoded by CTX-M, OXA, or AmpC β-lactamase (Paterson, 2006). Infections with ESBL-producing organisms place a strain on the healthcare system and are associated with mortality rates ranging between 3.7% and 22.1% (Gutierrez-Gutierrez et al., 2016). A report published in 2014 looked at healthcare-associated infections in 11,282 patients from 183 hospitals. The most frequent infections were found to be pneumonia (21.8%), surgical site infections (21.8%), gastrointestinal infections (17.1%), urinary tract infections (12.9%), and bloodstream infections (9.9%), with the leading causative organisms being *Clostridium difficile* (12.1%), *S. aureus* (10.7%), *Klebsiella* (9.9%), and *Escherichia coli* (9.3%) (Magill et al., 2014).

4. *Acinetobacter baumannii* is a Gram-negative opportunistic bacterium, commonly found in soil and water samples, and is a CDC pathogen that poses an urgent threat, with an estimated 8,500 infections and 700 estimated deaths per year (CDC 2022). It can contaminate and survive on the surface of the skin and medical devices, such as ventilator tubing, respirators, and arterial line pressure monitoring devices, for up to 27 days (Jawad et al., 1998). *Acinetobacter* infections are common in ICUs and can lead to prolonged exposure to antibiotics, increased ICU length of stay, and mortality rates ranging from 13% to 30% (Vincent et al., 2009)(Mathai et al., 2012)(Garnacho-Montero et al., 2015)(Hidron et al., 2008) (Garnacho-Montero et al., 2015). What makes *Acinetobacter* infections particularly dangerous and difficult to treat is their propensity for rapid and high-level development of resistance to most available antibiotics, including “last-resort” agents such as tigecycline, colistin, and polymyxins (Lee et al., 2017). *A. baumannii* possesses multiple intrinsic and extrinsic mechanisms of resistance that include a wide range of β-lactamases (e.g., TEM-1, CTX-M, OXA, and class B metallo-β-lactamases), defects in cell wall permeability porin channels, expression of multidrug efflux pumps, and alteration of target sites (PBPs and mutations in DNA gyrase). These mechanisms have to led to the isolation of MDR (resistance to three or more classes of antibiotics), extensively drug-resistant (XDR; MDR plus resistance to carbapenems), and pan-drug-resistant (PDR; XDR plus resistance to polymyxins) bacteria (Breijyeh et al., 2020). New treatment strategies must adapt to developing resistances, whether through the use of “old-school” agents like polymyxin, combination therapies with colistin and carbapenems, or the development of new therapeutic agents such as eravacycline and cefiderocol (Falcone et al., 2021).

5. *Pseudomonas aeruginosa* is a familiar nosocomial pathogen often found in ICUs across the USA. Although it has been isolated from normal gastrointestinal flora, it may also cause community-acquired and nosocomial infections in immunocompromised hosts (Chopra et al., 2008). Multidrug resistant *Pseudomonas* is a global health concern and a serious threat according to the CDC, with an estimated 32,600 cases and 2,700 deaths among hospitalized patients in 2017 (CDC 2022). Resistance to multiple classes of antibiotics is attained via intrinsic or acquired mechanisms, including the expression of β-lactamases (e.g., TEM, SHV, CTX-M, OXA, MBLs, and AmpC), target binding site alteration (e.g., 16S ribosomal RNA mutations, aminoglycoside-modifying enzymes, and *gyraA* and *parC* mutations), loss of surface porins such as OprD (conferring carbapenem resistance), and overexpression of efflux pumps capable of actively transporting antibiotics out of the cell (Fang et al., 2014). In addition to the mechanisms listed above, *Pseudomonas* has the ability to survive on devices found in healthcare settings (e.g., ventilators, urinary tract devices, and central line kits) through the formation of biofilm and persister cells (Hong et al., 2015). Prolonged exposure to anti-pseudomonal antibiotics has been reported to promote the selection and proliferation of MDR *Pseudomonas* and lead to high rates of colonization and prolonged duration of therapy in ICUs around the country (Raman et al., 2018).

6. *Enterobacter* is a genus of Gram-negative bacteria known to cause drug-resistant nosocomial infections. It has been isolated from the respiratory and urinary tracts, surgical wounds, cardiac and bone tissues, and blood samples of patients residing in ICUs. According to the Surveillance and Control of Pathogens of Epidemiologic Importance (SCOPE) report, *Enterobacter* was found to cause 4.7% of all ICU infections in US hospitals (Wisplinghoff et al., 2004). Particular attention is being paid to *Enterobacter aerogenes* and *Enterobacter cloacae* for their propensity to develop resistance to multiple antibiotics. All *Enterobacter* are naturally resistant to ampicillin, amoxicillin-clavulanic acid, and cefoxitin via the production of β-lactamases (Wisplinghoff et al., 2004). Moreover, they naturally express the AmpC β-lactamase, which is further inducible by exposure to broad-spectrum cephalosporins and has been found integrated into a large plasmid element capable of horizontal gene transmission (Lee et al., 2003). In addition, resistance to aminoglycosides and fluoroquinolones is acquired via intrinsic mutations or plasmid-encoded drug-modifying enzymes (Mezzatesta et al., 2012). Finally, more recent data show the emergence and spread of hospital-associated carbapenem-resistant *E. cloacae* across the USA, as well as the emergence of pan-drug-resistant strains, with resistance to colistin, around the world (Diene et al., 2013).

A summary of the mechanisms of MDR to antibiotics is presented in **Tables 1** and **2**.

**Table 1:** ESKAPE pathogens mechanisms of resistance.

Pathogen	Mechanisms of resistance
***Enterococcus faecium***	• β-lactamases • Cell wall modification • Aminoglycoside-modifying enzymes • Target enzyme modifications • Ribosomal target alterations • Porin alterations • Efflux pumps
***Staphylococcus aureus* (methicillin-resistant)**	
***Klebsiella pneumoniae***	
***Acinetobacter baumannii***	
***Pseudomonas aeruginosa***	
***Enterobacter* spp.**	

**Table 2:** Specific mechanisms of bacterial resistance to antimicrobial agents (ref. 11–12).

	Resistance mechanism	Examples of resistant genotype/phenotype	Conferred antimicrobial resistance
**β-lactamases**	Enzymatic degradation of the β-lactam ring via Ambler’s class β-lactamases: 1) Serine-type β-lactamases—class A, C, and D 2) Metallo-β-lactamases—class B	Class A (ESBLs)—TEM, CTX-M, KPC Class B—IMP, VIM Class C—AmpC Class D—OXA-48	Penicillins Cephalosporins Monobactams Carbapenems Emerging resistance to ceftazidime–avibactam
**Cell wall modification**	Modification of cell wall peptidoglycan precursor from D-Ala-D-Ala to D-Ala-D-lactate or D-Ala-D-serine Enzymatic removal of natural D-Ala-D-Ala precursors Alterations in lipopolysaccharide lipid A synthesis	*vanA, vanB, vancC* *mgrB, mcr, lpxA*, *IpxC, lpxD*	Vancomycin Daptomycin Emerging resistance to polymyxins
**Aminoglycoside-modifying enzymes**	Enzymatic degradation of aminoglycoside molecules via aminoglycoside acetyltransferases (AACs), phosphotransferases (APHs), and nucleotidyltransferases (ANTs), resulting in diminished activity	AAC(3), AAC(6′) APH(3′) ANT(4′), ANT(2″)	Aminoglycosides
**Target enzyme alterations**	Modification of peptidoglycan transpeptidases, namely the expression of a modified penicillin-binding proteins (PBP2a, PBP5) Modification of DNA gyrase and topoisomerase IV enzymes Plasmid-mediated quinolone resistance (PMQR)	*mecA, mecB, mecC* *gyrA, gyrB* QnrA, QnrB, QnrS	Ampicillin Methicillin Fluoroquinolones
**Ribosomal target alterations**	rRNA methyltransferase-mediated modification of bacterial ribosomal subunits (30S and 50S)	*erm(A), erm(B), erm(C)* *cfr*	Aminoglycosides Macrolides Linezolid
**Porin alterations**	Modification (e.g., upregulation, downregulation, loss of function) of outer membrane porins	OprD, Omp36, LamB	Penicillins Cephalosporins Monobactams Carbapenems Fluoroquinolones
**Efflux pumps**	Active efflux of antimicrobial agents via six major families: 1) Resistance–nodulation–division (RND) 2) Major facilitator superfamily (MFS) 3) Multidrug and toxic compound extrusion (MATE) 4) Small multidrug resistance (SMR) 5) ATP-binding cassette (ABC) 6) Proteobacterial antimicrobial compound efflux (PACE)	Mex-AB-OprM system AcrAB-TolC system OqxAB	Nearly all classes of antibiotics

ATP, adenine triphosphate.

## Prevention and treatment of antimicrobial resistance

### Antibiotic stewardship

Antimicrobial stewardship (AS) refers to an organized program designed to monitor, improve, and measure the responsible use of antibiotics in the ICU. It has emerged to overcome the risks of the inappropriate and inadequate use of antibiotics in the ICU. Inappropriate use of antibiotics describes the very early use of antibiotics with a too-broad spectrum, leading to an increased risk of resistant bacteria. Inadequate use of antibiotics describes the very early use of antibiotics that do not cover suspected organisms (Zilberberg et al., 2014).

Essentials of AS entail the following:

1. Facility-specific guidelines based on national guidelines and local antibiograms.
2. Restrictive programs in which a release of an antibiotic requires approval by an AS team member. This approval is based on cost, spectrum, and risk of side effects from the antibiotic of interest.
3. A persuasive program [also called prospective audit and feedback (PAF)] in which feedback in the form of modification or discontinuation of an antibiotic is provided by an AS team member on antibiotics that are prescribed in a special ICU.
4. Automatic stop orders in which there is a set date after which the prescribed antibiotics require an additional order to extend the duration of the prescription.
5. An antibiotic “time out” in which there is a prompt to review the prescribed antibiotics at a certain time point after initiation.
6. Rapid microbial identification using rapid diagnostic testing, such as real-time polymerase chain reaction (rtPCR), for the diagnosis of the genes of resistant bacteria such as MRSA from positive cultures.
7. Systematic de-escalation of antibiotics based on the kinetics of certain biomarkers, the most studied of which is procalcitonin.
8. Embedding a member of an AS team in ICU rounds.
9. Implementation of alerts in prescription software to guide clinicians at different stages of antibiotic prescription (Doernberg and Chambers, 2017).

### Antibiograms

An antibiogram is a snapshot of microbial susceptibility to various antibiotics reported over time. It can provide useful insights into current resistance patterns and past and future trends, and help guide empiric antibiotic treatment. Antibiograms are commonly encountered in a table format that categorizes bacteria as Gram positive or Gram negative and breaks them down further by individual species. Susceptibility rates to commonly used antibiotics are reported as percentages. Depending on facility capabilities, antibiograms can drill down into specific susceptibilities based on patient location, such as ICUs, and sites of infections, such as blood, sputum, or urine. Susceptibility patterns can also have intrahospital variability. A study by Kaufman et al. compared hospital-wide susceptibility data with isolates from surgical intensive care unit (SICU) patients. The two most commonly isolated organisms were *S. aureus* and *P. aeruginosa*. Surprisingly, the susceptibility patterns were significantly different between the hospital-wide and SICU data (Kaufman et al., 1998). Another study by Chang et al. (2023) evaluated the effect of empiric antibiotics on mortality in patients with bloodstream infections in the emergency department (ED). They found that empiric antibiotic selection based on a local antibiogram was associated with a reduced mortality rate and a shorter length of stay in the ICU (Chang et al., 2023).

Antibiograms are a useful tool in the clinician’s arsenal to help prevent the emergence of antimicrobial resistance, but they also have some limitations. First, antibiogram reporting is binary, indicating whether or not a specific isolate is susceptible, and does not account for minimum inhibitory concentrations (MICs). The Clinical and Laboratory Standards Institute (CLSI) publishes an annual report with MICs for various bacterial pathogens. According to the 2023 CLSI report, *S. aureus* with a vancomycin MIC of ≤ 2 µg/ml is considered susceptible (“M100” n.d.). Consequently, every *S. aureus* isolate with that MIC will be reported as susceptible on an antibiogram. Despite the fact that an antibiogram is technically accurate, it is recommended to avoid using vancomycin to treat *S. aureus* isolates with an MIC of 2 µg/mL or greater (Rybak et al., 2009). Second, antibiograms may provide false information regarding the timing of cultures, which may lead to erroneous diagnosis of time-dependent classifications, such as community-acquired pneumonia (CAP) compared with hospital-acquired pneumonia (HAP). This in turn may lead to inappropriate use of antibiotics, as the treatment for CAP and HAP is different and aims at covering different pathogens based on indication.

In summary, antibiograms are an important tool in helping ICU practitioners select appropriate empiric antibiotic regimens that will provide a broad coverage and simultaneously help curtail the development of antimicrobial resistance. They also come with important limitations and are meant to be used as an aid and not a replacement for clinical judgment or multidisciplinary collaboration with members of the ICU team, including physicians, mid-level practitioners, and pharmacists.

### Surveillance

Surveillance entails monitoring trends in the susceptibilities of microorganisms isolated in one or more ICUs within a hospital, identifying patients colonized or infected with MDR bacteria, and monitoring trends in the use of antibiotics. Practices vary in terms of the population of patients to undergo surveillance, the laboratory assays used to perform surveillance, the ICUs put under surveillance, and the microorganisms surveilled. In addition to the time and expense of routine universal ICU surveillance, there is mixed evidence on whether or not it truly guides initial antibiotic therapy because of its low sensitivity (Hayon et al., 2002; Sanders et al., 2008).

### Pharmacokinetic/pharmacodynamic optimization

Alterations in renal function and flow, ranging from augmented renal clearance to acute kidney injury (AKI) and failure requiring the initiation of renal replacement therapy, have strong implications for antibiotics that are renally cleared, such as β-lactams. Patients with augmented renal clearance may need an increased initial dosage or infusion of β-lactam antibiotics to achieve adequate tissue levels. On the other hand, patients with AKI may need renal dose adjustments. An aggressive pharmacodynamic (PK) approach, though not a standard of care at present, may be needed for novel β-lactam antibiotics to reduce resistance (Gatti and Pea, 2021).

### Adequate infection control measures

Adequate infection control measures in the form of standard precautions (hand hygiene, gloves, gowns, and eye protection) in addition to transmission (i.e., droplet, airborne, or contact)-based precautions were developed to reduce the transmission of resistant microorganisms from one site to another. Adherence to isolation precautions has been conclusively shown to reduce the transmission of MRSA and extended-spectrum β-lactamase (ESBL)-producing *Enterobacteriaceae* (Bassetti et al., 2015).

### Adequate source control

Source control is the removal of the source of infection by the drainage of pus or inflammatory material, or debridement of necrotic tissue, and is especially important in surgical patients. Achieving early source control is associated with a reduction in antibiotic duration and resistance. Timing of source control should be guided by the severity of infection, source of infection, and hemodynamic stability of the patient (Kisat and Zarzaur, 2022). Successful signs of source control include, but are not limited to, the resolution of clinical signs of infection, such as fever and leukocytosis, the amelioration of tenderness, radiological resolution in the form of reduction in the size of infected fluid, and the disappearance of previously recognized fistulas or abscesses and a reduction in drainage from tubes and drains (Solomkin et al., 2013).

## Conclusion

Antibiotic resistance remains an ongoing challenge for patients admitted to the ICU. The imbalance between limited antibiotic pools on one side and rapidly evolving microbial resistance on the other warrants attention and the implementation of more rigorous preventive strategies, such as rapid diagnostic testing, AS policies, strict infection control measures, and PK/PD drug dosing and monitoring. Novel areas of research, such as studying the interactions of antibiotics and gut microbiota using metagenomic techniques, the protection and restoration of the commensal gut ecosystem via fecal transplantation or orally administered probiotics (Ruppe et al., 2018), and the novel aerosolized routes for administration of antibiotics for lung infections (Montgomery et al., 2014), are urgently needed in the current battle against antimicrobial resistance.

## Author contributions

AZ developed the concept and the manuscript design, and wrote and edited the final manuscript draft. RK and DS participated in manuscript design and wrote the initial manuscript draft. All authors contributed to the article and approved the submitted version.
